# Supplementary material for: Unveiling CNS cell morphology with deep learning: A gateway to anti-inflammatory compound screening
Source: PLoS One. 2025 Mar 21;20(3):e0320204. doi: 10.1371/journal.pone.0320204 (PMC11927906; doi:10.1371/journal.pone.0320204)
Supplement: S1 Appendix — (DOCX) [file pone.0320204.s001.docx]

**S1 Appendix**

During the model development phase, our primary focus was on assessing the accuracy of the model to classify images of primary cultured cells based on varying LPS concentrations. To achieve this objective, our meticulous approach involved a thorough 4-fold cross-validation process to pinpoint the optimal model for our specific task. In this rigorous evaluation, one model stood out with an exceptional accuracy of 96%, surpassing the performance of the other models by a substantial margin of approximately 5% (figure A in S2 Fig). Such superior performance highlights not only the model's robustness but also its remarkable predictive capabilities, making it the clear choice for our intended application. The selection of this top-performing model ensures the reliability and accuracy of our results, instilling confidence in the validity of our findings and the effectiveness of our approach.

Transitioning to the final test, our focus shifted towards investigating the practical applicability in a pharmacological context by measuring how well our model predicts the efficacy of known bioactive compounds. This evaluation was quantified through the ratios of cell images classified as control across increasing anti-inflammatory compounds. Furthermore, this phase prompted a critical examination of the conventional paradigm of deep learning, which involves selecting the best-performing models based on cross-validation. When dealing with cell image data, it became evident that this approach had limitations due to the presence of batch effects that could significantly influence the final test outcomes: the best model showed poor performance in predicting the dose-dependent efficacy (figure E in S2 Fig). Notably, the ensemble model (figure F in S2 Fig) demonstrated that the control ratios increased in a dose-dependent manner when exposed to increasing doses of the anti-inflammatory compounds. To quantify the evaluation result, we calculated Pearson correlations between the control probabilities and the doses of the three anti-inflammatory compounds. Higher correlation coefficients indicate better performances in predicting dose-dependent efficacy. The detailed examination revealed significant variability in the correlation across the different models. However, the ensemble approach consistently outperformed the individual models (Dimethyl Fumarate or DMF) or closely matched the best individual model’s performance (Resatorvid, Dexamethasone), effectively mitigating the uncertainty inherent in relying on a single best model (figure G in S2 Fig). Therefore, adhering to the conventional approach and selecting the best model (figure A in S2 Fig) would result in less accurate and potentially biased predictions. This ensemble approach not only enhances predictive accuracy but also contributes to the overall stability and reliability of our predictions. By considering the diverse characteristics of different plates and accounting for the intricacies of batch effects, ensemble learning emerges as a robust solution for achieving more stable and accurate predictions. Taken together, ensemble learning is an effective strategy in our study, enabling us to overcome the challenges posed by batch effects and reliably predict cellular states with improved accuracy and stability.

S3 Fig displays feature maps derived from the convolutional layer of EfficientNet-B5, which are trained to capture features of various cellular phenotypes such as nucleic acids (blue), neurons (green), and microglia (red). The characteristics of these feature maps include:

Nucleic Acid Feature Maps: These maps show a high contrast between regions of varying importance, with a multitude of features depicted. The clear distinction of various features, transitioning from subtle to prominent, may contribute to increased model complexity.

Neuron Feature Maps: These maps exhibit a more uniform color distribution, reflecting ambiguity in feature importance. The lack of clear separation makes it challenging to discern which features are significant, potentially leading to computational inefficiency.

Microglia Feature Maps: Compared to other types, these maps have fewer features, yet with a very distinct weight distribution. This clear differentiation helps in easily identifying important regions, likely enhancing model accuracy and predictive performance.

S4 Fig presents feature maps from the activation layer of EfficientNet-B5. These maps highlight the regions deemed significant by the model for different cellular phenotypes:

Nucleic Acid Feature Maps: The contrast between highly important and less important regions is visually apparent, with essential features of nucleic acids highlighted effectively.

Neuron Feature Maps: These maps may show a blend of well-learned and poorly learned features, indicating a balanced but complex representation of neuronal structures.

Microglia Feature Maps: Feature maps of microglia demonstrate a clear and concise distribution of features, aiding in straightforward interpretation of the model's focus.

Merging these feature maps into a single three-channel image (R, G, B) allows for an efficient arrangement of features, capitalizing on the strengths of each map while addressing their limitations. This method offers a comprehensive view, enhancing the overall accuracy and model's ability to make precise predictions.

By modifying the content in this way, you align the descriptions with the specific nature of the feature maps from the convolutional and activation layers, providing a clearer and more detailed understanding of the model's behavior in feature detection and importance weighting.
