# Supplementary figures and images for: Unveiling CNS cell morphology with deep learning: A gateway to anti-inflammatory compound screening
Source: PLoS One. 2025 Mar 21;20(3):e0320204. doi: 10.1371/journal.pone.0320204 (PMC11927906; doi:10.1371/journal.pone.0320204)

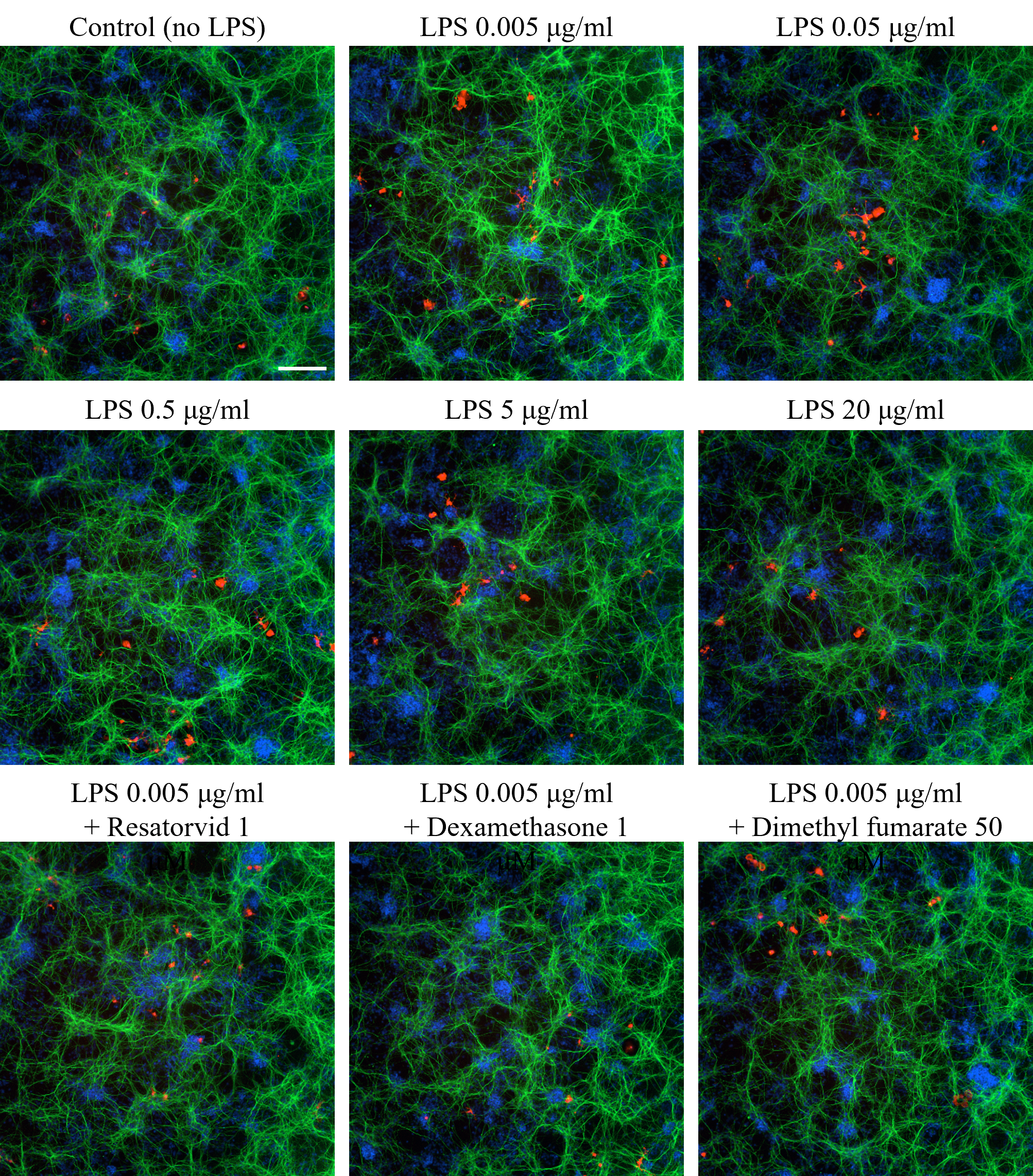

Supplement: S1 Fig — MAP-2 (neuron, green), and DAPI (nucleic acids, blue). The first and second rows show the cells treated with increasing concentrations of LPS. The third row displays the cells exposed to LPS and a range of anti-inflammatory compounds, providing a comparative perspective on the effectiveness of these compounds. (TIF) [file pone.0320204.s002.tif]

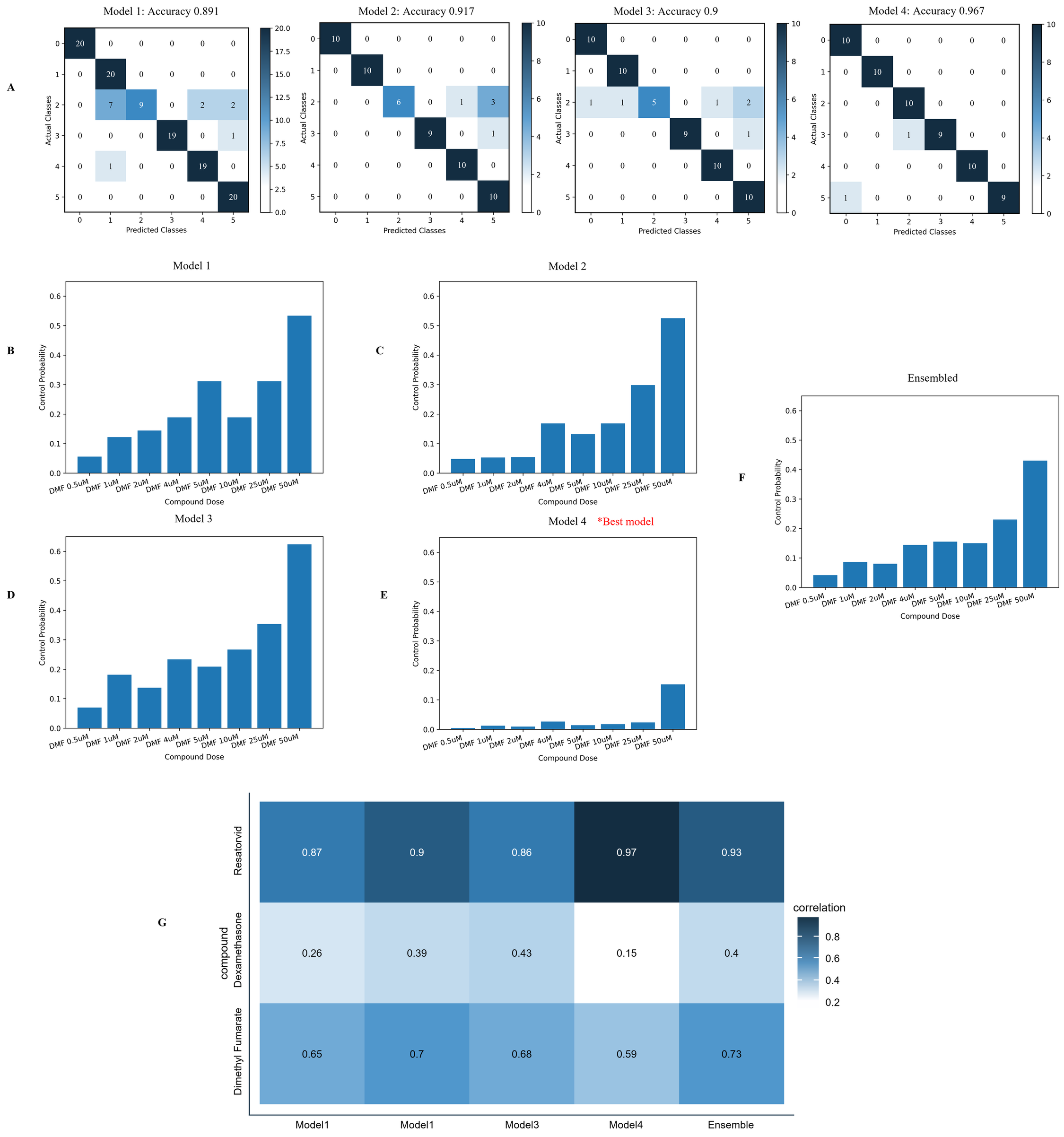

Supplement: S2 Fig — (A) The confusion matrix from the model deployment step illustrates how accurately four models classify the LPS concentration given to the primary cultured cells. The diagonal entries of the matrix represent the number of the correctly classified wells of plates. (B-F) In the efficacy test, ratios of cell images classified as control are expressed as a function of increasing concentrations of an anti-inflammatory compound. The ratios for 4 models (B-E) derived from 4-fold cross-validation alongside the ensemble model (F). (G) A heat map of Pearson correlations between the control probabilities and the doses of the three anti-inflammatory drugs administered to the primary cultured cells. (TIF) [file pone.0320204.s003.tif]

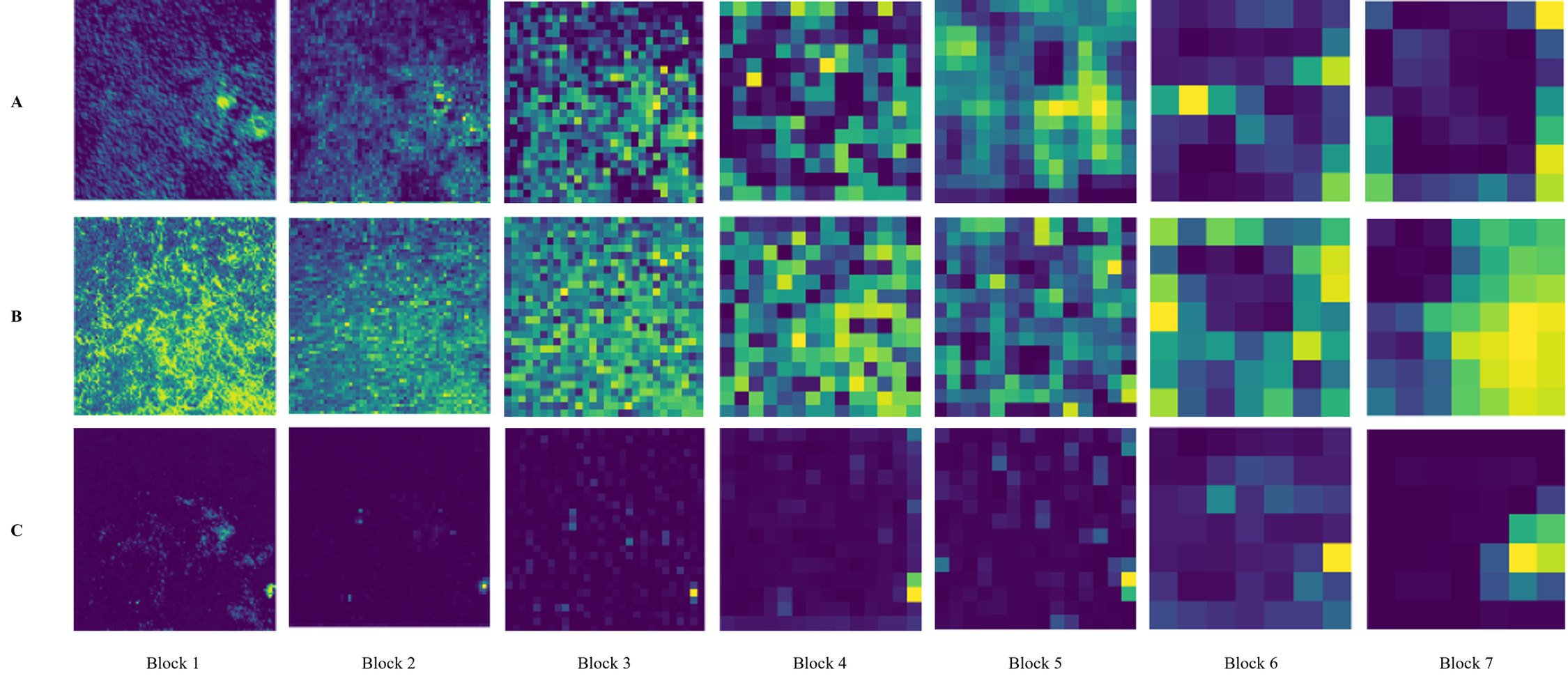

Supplement: S3 Fig — This figure presents feature maps extracted from the Convolutional Layer for various cellular properties. The Convolutional Layer captures low-level features in images, and this figure visualizes the structural characteristics identified for each cellular marker. In the context of the viridis colormap, which is often used for this purpose, the spectrum typically ranges from purple to yellow. Purple or dark colors represent areas with lower weights or less significance in the feature map, indicating regions that the model does not consider important for its predictions. On the other hand, yellow or bright colors correspond to higher weights or more important regions, showing features that the model heavily relies on for its decision-making process. (A) Nucleic acids (blue): The map emphasizes subtle features such as nuclear structures and arrangements within cells, clearly differentiating areas with and without nucleic acids. (B) Neurons (green): The complex morphology and network of neurons are reflected, with distinct visualization of cell bodies and dendritic structures. (C) Microglia (red): The unique shape and distribution of microglia are highlighted, visualizing structural changes based on the cell’s activity state. (TIF) [file pone.0320204.s004.tif]

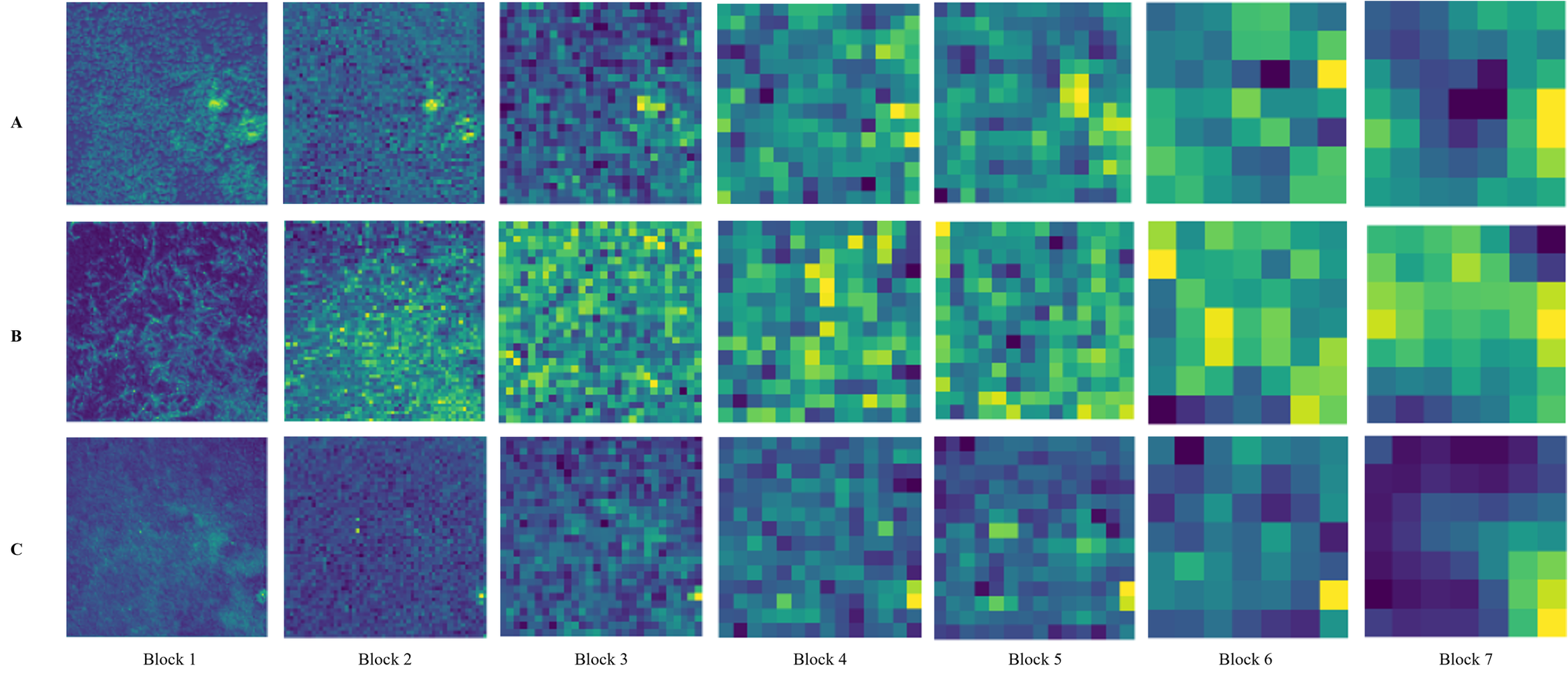

Supplement: S4 Fig — This figure demonstrates feature maps from the Activation Layer, illustrating which regions the model deems significant. Using the viridis colormap, areas of importance are differentiated by color, with each feature map showing the following characteristics. (A) Nucleic acids (blue): There is a clear distinction between important and less significant regions, with vital parts of nucleic acids highlighted in brighter colors. (B) Neurons (green): The map emphasizes significant structures of neurons, though the differentiation of importance may be less distinct. (C) Microglia (red): Key areas of activated microglia are brightly colored, effectively revealing important features. (TIF) [file pone.0320204.s005.tif]
