# Supplementary material for: Unveiling CNS cell morphology with deep learning: A gateway to anti-inflammatory compound screening
Source: PLoS One. 2025 Mar 21;20(3):e0320204. doi: 10.1371/journal.pone.0320204 (PMC11927906; doi:10.1371/journal.pone.0320204)
Supplement: Attachment — Submitted filename: Response to Reviewers.docx [file pone.0320204.s008.docx]

We thank you and the reviewers for your careful evaluation of our manuscript ***“Unveiling CNS cell morphology with deep learning: A gateway to anti-inflammatory compound screening”***. We appreciate the constructive feedback, which has helped us improve our manuscript significantly.

We have thoroughly addressed all the comments and revised our manuscript accordingly. Below, we provide a point-by-point response to each reviewer's comments.

***Reviewer #1:***

***Comment 1:***

While the model development process is very well-articulated, the model evaluation portion is less clear. Namely, the paragraph beginning on line 209 references two confusion matrices (Figure 3c and 3d) implying a testing paradigm on 5 wells per class (Fig 3d) and their constituent 15 cropped images for a total of 75 image-wise predictions per class (Fig 3c). It is unclear where these 5 wells were selected from (i.e., from the held-out validation fold?). Lines 251-261 under section “Ensemble-based batch effect mitigation” mentions “predicting one of the 15 cropped images generated from a single well” which does not help in clarifying the confusion regarding the model testing procedure.

***Response to Comment 1:***

Thank you for pointing out the need for clarity in our model evaluation procedure.
The confusion matrices in Figure 3c and 3d were generated using the best performing model among the four EfficientNet-B5 models trained during our leave-plates-out cross-validation process. Specifically, these results came from the validation case where the validation set contained one plate.

For this single validation plate:

1. Image-level predictions (Figure 3d):
   - Contains 450 images total (5 wells × 15 cropped images × 6 classes)
   - Shows how individual cropped cell images were classified
2. Well-level predictions (Figure 3e):
   - Contains 30 wells total (6 classes × 5 replicate wells)
   - Represents how these wells were classified based on their inflammatory states
   - Each well's classification is determined by aggregating predictions from its 15 cropped images

We have revised the Results section (line 245) to clearly explain this evaluation process and its relationship to our cross-validation procedure.

***Comment 2:***

Figure 3 on Line 175 seems to have replicated subfigures c-e.

***Response to Comment 2:***

Thank you for bringing this to our attention. Upon checking, we found that subfigures c-e were indeed mistakenly replicated in Figure 3. We have corrected Figure 3 to show each subfigure only once.

***Comment 3:***

Regarding the leave-one-plate-out cross-validation, the authors chose not to select an optimal validation model and instead combine all trained models with an ensemble based-strategy. They claim that this reduces batch-like effects from the plates. However, it is unclear why the authors chose to still group plates together into 4 folds (combining two plates per fold except the final fold which includes only one plate) and not instead run 7 trials with 7 folds corresponding to each distinct plate.

***Response to Comment 3:***

Thank you for this insightful question about our cross-validation design.

While using 7 folds (one for each plate) might appear more intuitive, we opted for 4 groups to optimize computational efficiency. This approach reduced computational overhead by training fewer models while still effectively capturing plate-to-plate variations. The reduced computation time is particularly crucial for practical drug screening applications, where rapid processing of large datasets is essential.

***Comment 4:***

The dose-dependent effects of anti-inflammatory compounds, particularly in microglial cells is impressive. However, the inability of the model to predict controls as such in the nucleus and neuron channels is concerning (<50%). Further explanation from the authors regarding the poor performance in these cell types, especially with the non-inflamed states, would benefit the discussion section of the manuscript. The authors claim themselves that “The near-uniform distributions of feature importance in the other two maps (neurons and nucleic acids) without emphasis on certain areas complicated the task of identifying which aspects of the cell images were critical to the prediction” (lines 358-360), yet continue to support the stance of combining the three images of cellular markers into a unified image, despite most of the contributions seemingly coming from the microglial cells (i.e., microglia-channel only models seem to show anti-inflammatory efficacy more clearly than the 3-channel models in Figure 5). A more thorough discussion on what the other two cell types contribute may help strengthen this stance.

***Response to Comment 4:***
Thank you for these insightful observations. We have added more explanation of the multi-channel integration to the Discussion section (line 497).

***Comment 5:***
Regarding data preprocessing, the authors highlight the impacts of preprocessing parameters on model performance (lines 492-496) but do not explain further how these parameters were selected. A section in the supplementary data addressing this parameter selection would be beneficial.
***Response to Comment 5:***
Thank you for raising this important point about our preprocessing parameter selection. We added explanations of each preprocessing method and its intended purpose. The unsupported claim about preprocessing impacts on model performance was removed (line 201). We followed standard preprocessing approaches rather than conducting systematic parameter optimization studies, so we chose not to add a supplementary section.

***Comment 6:***

Overall, the authors’ undertaking of an intensive comparative study on 14 different models with two very relevant architectures (CNNs and ViTs) is commendable. The conclusions support the well elucidated rationale for phenotypic screening of anti-inflammatory compounds in the context of pharmacological testing. Only a few methodological clarifications are needed before publication.

***Response to Comment 6:***
We have added more explanations to Discussion.

***Reviewer #2:***

***Comment 7:***

-Figure 1: You label part A with "Test image dataset" but call it the "Validation set" in part B, as well as in the rest of the document. I would change the former to say Validation to be more consistent, because you do not have both a Validation set and a Test set.

***Response to Comment 7***

Thank you for noting this terminology inconsistency. We have revised the label from "Test image dataset" to "Validation set" to maintain consistent terminology throughout the manuscript and figures.

***Comment 8:***

-Line 114: Can you specify the exact size of the training set? It is too vague to say "thousands" and would be helpful for context as to how many are in each class and why you would need augmentation.

***Response to Comment 8:***

We have integrated the following description of the number of samples (image-level, well-wise) to the method section (line 577) and clarified the reason behind using augmentation to “Transforming Cellular Microscopic Images for Deep Learning: Staining, Augmentation, and Normalization Techniques” section in Method.

Dataset Composition:

We used a total of 3,150 cellular images collected from seven experimental plates to train CNN and ViT models. Each plate contained six experimental classes: one control group and five groups treated with different LPS concentrations. For each class, we used five replicate wells, with each well generating 15 cropped images. Thus, each plate yielded 450 images (6 classes × 5 wells × 15 crops = 450 images), resulting in a total dataset of 3,150 images across all seven plates (450 images × 7 plates).

Cross-validation Design:

To ensure robust model validation while accounting for plate-specific effects, we implemented a modified leave-one-group-out cross-validation strategy. The seven plates were systematically combined into four groups. Three groups were formed by combining two plates, where one group was left with a single plate. During cross validation, one group served as the validation set while the remaining group formed the training set. This approach ensured that cell images from the same plate remined together in the same fold, preventing data leakage. The validation set contained either 900 images when using groups 1-3 (2 plates × 450 images per plate) or 450 images when using group 4 (1 plate × 450 images).

Well-wise prediction:

For well-wise predictions, we used the data at the well level across our experimental dataset. Each plate contained 30 wells, comprised of 6 classes (one control and five LPS concentrations) with 5 replicate wells per class. Across all seven plates, this yielded a total of 210 wells (30 wells × 7 plates).

***Comment 9:***

-Line 167: Why was only 10% of the dataset augmented?

***Response to Comment 9:***

Thank you for this important question about our data augmentation strategy. While augmentation is beneficial for mitigating overfitting, excessive augmentations may introduce noises that diverge from biological characteristics of the original data, potentially impacting model performance. We acknowledge that our choice of 10% was not determined empirically. However, by limiting augmentation to this level, we aimed to diversify the data while minimizing the risk of over-generalization. Given that this choice yielded good model performance, we did not need to empirically determine the optimal augmentation.

We have added the following explanations (line 188) to clarify their intended purpose

““Approximately 10% of the dataset was augmented using shift, rotation, flip, enlargement, and reduction of image coordinates, to increase dataset size and variability (Fig 2c, d). This level of augmentation was selected to balance enhancing the model’s robustness and preserving the integrity of the original data. While augmentation is beneficial for mitigating overfitting, excessive augmentations may introduce noises that diverge from biological characteristics of the original data, potentially impact model performance. By limiting augmentation to 10%, the data was diversified while minimizing the risk of over-generalization.”

***Comment 10:***

-Line 172: Can you quantify what the positive effect of preprocessing on the accuracy was?

***Response to Comment 10:***

Thank you for this important question. While we applied conventional preprocessing methods that are widely established in deep learning literature to improve model generalization, we acknowledge that we did not conduct studies to quantify the specific impact of each preprocessing step on our model's accuracy. This is a valuable suggestion for future work, where systematic evaluation of each preprocessing component's contribution could provide insights into their relative importance for cell morphology analysis.

We have removed the claim about the positive effects on accuracy (line 201)

Thank you for this suggestion.

***Comment 11:***

Figure 3: In my version, 3C-E are duplicated. Please remove one set.

***Response to Comment 11:***

We have fixed it. Thank you.

***Comment 12:***

-Line 203: Is the accuracy based on the image task or the well task? It is not explained here. Are different models better for different tasks?

***Response to Comment 12:***

The accuracy reported in our study is primarily based on the image-level prediction, which serves as an intermediate step toward well-level prediction. Specifically, the model predicts the class for each cropped image, and these predictions are then aggregated to determine the final prediction for the corresponding well.

The ultimate goal of our study is to accurately predict the state of each well, as this is critical for evaluating the drug’s efficacy. Well-level prediction provides a more robust and interpretable assessment of drug effects because it reflects the overall state of the cellular population within a given well, rather than individual images, which may exhibit variability.

Regarding the suitability of different models for different tasks, our findings indicate that the same model architecture can be effective for both image-level and well-level predictions when paired with an appropriate aggregation strategy. By using the ensemble method and the CTL proportion-based approach, we successfully bridged the gap between these two levels, achieving robust performance in well-level prediction.

The following are several benefits of well-wise predictions particularly for drug screening applications.

1. Enhanced Robustness: Well-wise predictions capture the collective state of cellular populations by aggregating multiple images per well, smoothing out variability in individual image predictions and better reflecting the biological reality of non-uniform cellular responses.
2. Improved performance: In Figure 3e, our model demonstrated superior classification accuracy across all pathological states when using well-wise predictions compared to image-wise predictions (Figure 3d).
3. Since compounds are administered at the well level, well-wise predictions correspond better with actual drug screening. This approach minimizes inaccurate predictions that could lead to significant costs in drug discovery processes.
4. Batch effect mitigation: The considerations of multiple images per well for a final prediction helps account for plate-to-plate variations.
5. Quantitative assessment: This quantitative assessment provided better insights into the degree of drug response by using the percentages of control cells in well.

***Comment 13:***

-Line 206: Typo "CNNs models" -> "CNN models"

***Response to Comment 13:***

We have fixed it.

***Comment 14***

-Figure 4: Typo "Model devlopment set" -> "Model development set"

***Response to Comment 14:***

Thank you for catching this typographical error. We have corrected "Model devlopment set" to "Model development set" in Figure 4 legend.

***Comment 15***

-Line 240: It is more rigorous to say that it is a form of stratified cross-validation because you have intentionally split your data to be ordered in some way. I would mention that is also a standard method of splitting for cross-validation in addition to random.

***Response to Comment 15:***

Thank you for this suggestion. While stratified cross-validation maintains similar class distribution across all folds, our approach follows a different principle - keeping all data from a single plate together in the same fold. This plate-based strategy was designed to reflect real-world conditions where new plates would be tested on models trained on different plates. While each plate in our experimental setup naturally contains a balanced class distribution, this balance comes from the experimental design itself, not from our splitting strategy which, unlike stratified methods, makes no attempt to maintain preserve the percentage of samples for each class. Our method is conceptually like “Leave One Group Out cross-validator” of a machine learning library (https://scikit-learn.org/1.5/modules/generated/sklearn.model_selection.LeaveOneGroupOut.html). The method splits data such that each training dataset consists of all samples except ones belonging to one specific group. In our case, plate information is provided as an array of integers that encodes the group of each sample. So, cell images from the same group (a plate or group of plates) stay together.

We have made two specific changes to improve precision:

1. We have revised the "Ensemble-based batch effect mitigation" section of Results to better explain our cross-validation approach
2. We have updated the terminology from "leave-one-plate-out cross-validation" to "leave-plates-out cross-validation" throughout the manuscript to more accurately reflect our methodology of using plate groups

***Comment 16:***

-Line 258: There is confusion because you suddenly introduce this binary variable R when you previously had a classification with multiple classes. It is not explained until the next section why this is the case, so you may want to include some of that background after you introduce R.

***Response to Comment 16:***

Thank you for pointing out this lack of clarity in our transition to binary classification. We have added the following explanation to the Discussion section (line 485) to clarify our rationale for using binary variables. “Our classification strategy employs distinct approaches for the model training and final compound screening. During training, the model was tasked with classifying six cellular states, enabling it to learn a comprehensive spectrum of inflammatory states and their corresponding morphological features. However, for the final compound screening phase, we adjusted the model to generate binary predictions (control vs. LPS-treated) and quantified the result as the percentages of cells predicted to be in a control state. This binary evaluation corresponds with the main goal of drug screening: determining whether a compound can restore the inflamed cells to control-like cells. Expressing the predictions as a percentage provides a quantitative and effective metric for assessing drug efficacy, facilitating the establishment of dose response relationship. ‘This strategic transition from multi-class training to quantitative evaluation via binary predictions leverages the model’s ability to discern subtle morphological changes.”

***Comment 17:***

-Line 275: If the ultimate goal was to train a model for binary classification, why first train models to classify various degrees? It is not well explained here.

***Response to Comment 17:***

We have added an explanation to Discussion (line 485).

***Comment 18:***

-The Discussion section reads more like a Conclusion section. It would be helpful to distinguish the Discussion from the Conclusion here.

***Response to Comment 18:***

Thank you for this helpful observation.

We have enhanced the Discussion section by adding the following:

1. Additional interpretation of our approach (line 485)
2. Current limitations of our approach (line 495)
3. Discussion of potential future research direction (line 495)
4. The final paragraph serving as our conclusion (line 513)

***Comment 19:***

-Line 321: Typo "CNNs-based models" -> "CNN-based models"

***Response to Comment 19:***

Thank you for identifying this typo. We have made the suggested correction throughout the manuscript to maintain consistency.

***Comment 20:***

-Line 328: Earlier, you noted 88% as the accuracy, but now it is noted as the F1 score. Later in the Methods section, you distinguish between accuracy and F1 score, so which one is actually 88%? And why was the F1 score not mentioned earlier***?***

***Response to Comment 20:***

Thank you for catching this inconsistency. We confirm that 88% refers to the F1-score, not accuracy. We have revised the manuscript to consistently use F1-score throughout.

***Comment 21:***

-Line 329: You mention the importance in the multi-class task, but the Results focused on the binary classification. The message is a little bit unclear to me.

***Response to Comment 21:***

Thank you for pointing out this lack of clarity regarding our classification approach. We have added a detailed explanation to the Discussion section (line 485) to clarify our strategic transition from multi-class to binary classification.

***Comment 22:***

-Line 338: Again, it is not modified cross-validation, just stratified.

***Response to Comment 22:***

Thank you for this important clarification about our cross-validation terminology. We have revised the "Ensemble-based batch effect mitigation" section of Results to more accurately describe our approach.

***Comment 23:***

-Line 339: Based on your approach, it is more rigorous to say multiple models each generated a prediction, rather than each model generating multiple predictions.

***Response to Comment 23:***

Thank you for this precise correction in describing our ensemble approach. We agree that "multiple models each generated a prediction" more accurately reflects our methodology than "each model generating multiple predictions." We have revised this phrasing on line 455 to ensure technical accuracy in describing our ensemble prediction process.

***Comment 24:***

-Line 344: Where is this the prevailing notion? I feel most people in the machine learning field would not jump to such a conclusion.

***Response to Comment 24:***

Thank you for this important feedback. We have removed this unsupported claim from line 460.

***Comment 25:***

-Line 355: Why are those figures in Supplementary rather than in the main text? They seem important to note.

***Response to Comment 25:***

-The feature map analysis illustrates the extracted patterns of different cellular phenotypes from the convolutional layer. While these insights are valuable for understanding how the model processes the data, they were not directly used to generate the prediction results presented in the main text.

Instead, these analyses were included in the Supplementary Figures to provide additional context for readers who are interested in exploring the underlying features that the model identifies. We believe this analysis has significant potential to inform future research and further investigations into the biological basis of predictions. However, as they were not directly applied in generating the primary results, we decided to place them in the Supplementary section rather than in the main text.

This distinction ensures that the main text focuses on the core findings and methodologies directly tied to the study's objectives while providing supplementary material for readers interested in deeper exploration.

***Comment 26:***

-Line 373: Typo "CNNs-based algorithm" -> "CNN-based algorithm"

***Response to Comment 26:***

Thank you for identifying this typographical error. We have corrected "CNNs-based algorithm" to "CNN-based algorithm".

***Comment 27:***

-Line 499: It does not contribute because it mentions but does not quantify the benefits on accuracy.

***Response to Comment 27:***

We have removed the claim (line 648~ 651) for clarity. Thank you for this suggestion.

***Comment 28:***

-Did you consider the classification confidence level? For example, in a binary case, does your model return a probability of the classes, and are some of these inferences more confident than others?

***Response to Comment 28:***

- In our study, we did consider the classification confidence level by utilizing the prediction probabilities generated by the model. Specifically, we experimented with a confidence-based approach, where the predicted probabilities for each class were used to assign weights to the predictions, effectively adjusting the final outputs based on the confidence levels.

However, this approach did not yield better results compared to our current strategy. One possible reason for this outcome is that the inherent variability in our dataset, particularly at the image level, may introduce noise into the confidence scores, reducing their reliability for final predictions. In contrast, our current approach, which aggregates predictions at the well level based on the CTL proportion, effectively minimizes such noise and provides a more stable and interpretable evaluation of the drug's efficacy.

While classification confidence remains an interesting parameter to explore further, our results suggest that it may not be the optimal metric for this particular dataset and task. This insight has been added to the manuscript to address this aspect explicitly.

***Reviewer #3:***

***Comment 29:***

The manuscript discusses the use of CNN and ViT for classifying inflammatory states in CNS cells. While EfficientNet-B5 is selected as the optimal model, the criteria and reasoning behind the preference over ViT models could be further clarified. Including more detailed comparisons, particularly around model interpretability and applicability for CNS cell morphology, could enhance the reader’s understanding.

***Response to Comment 29:***

Thank you for this valuable suggestion to clarify our model selection rationale. We have strengthened our discussion of the architectural differences between CNN and ViT models by integrating the following explanation to the Discussion section (second paragraph):

“Our comparison of CNN and ViT models revealed distinct architectural advantages in processing cellular features. While ViTs excel at capturing global patterns and overall image context, CNNs proved more effective at learning the fine, hierarchical details crucial for analyzing microglia cells. This is particularly important as microglia exhibit diverse phenotypic states characterized by branching patterns, and cell body shapes. The superior performance of CNNs (Fig 3c) can be attributed to their convolutional filters being especially well-suited to capturing the hierarchical nature of cellular features and detecting detailed cellular structures, whereas ViTs, despite their strength in understanding overall patterns, showed limitations in capturing such fine-scale features.”

***Comment 30:***

How many images are in the training and test set? I suggest that the authors clearly state the number *of training and test samples and the train-test-split ratio.*

***Response to Comment 30:***

We have added the following paragraph to the "Image acquisition" section in Methods, directly after "Nuclei, MAP2, and Iba-1 images were acquired using DAPI, GFP, and Texas Red® filter channels, respectively." (line 577):

“We used a total of 3,150 cellular images collected from seven experimental plates to train CNN and ViT models. Each plate contained six experimental classes: one control group and five groups treated with different LPS concentrations. For each class, we used five replicate wells, with each well generating 15 cropped images. Thus, each plate yielded 450 images (6 classes × 5 wells × 15 crops = 450 images), resulting in a total dataset of 3,150 images across all seven plates (450 images × 7 plates).”

We have added the description of *the train-test-split ratio* to “Ensemble-based batch effect mitigation”.

Thank you for this suggestion.

***Comment 31:*** The confusion matrix in Figure 3 is not very clear. Which model is used to generate the plot and why are there few sample sizes?

***Response to Comment 31:***

Thank you for this important observation about the confusion matrices in Figure 3.

Model Selection:

These confusion matrices were generated using the best performing model among the four EfficientNet-B5 models trained during Leave-Plates-Out Cross-Validation process. In our cross-validation setup, seven plates were grouped into four groups, where each group contained either two plates or one plate. From the four models generated through our cross-validation process (each validated on a different group), we selected the best performing model, which came from the validation case where the validation set contained one plate.

Sample Size Representation:

1. Figure 3D (Image-level):
   - Represents predictions for 450 images (5 wells × 15 cropped images × 6 classes) from the single validation plate
   - Each cell in the matrix shows the number of individual cropped cell images classified into respective categories
2. Figure 3E (Well-level):
   - Presents results from the same validation plate at a well-wise level
   - Contains data from 30 wells (6 classes × 5 replicate wells)
   - Classes include one control group and five different LPS concentrations
   - Numbers indicate how these 30 wells were classified based on their inflammatory states

The seemingly small sample size in the well-wise predictions (30 wells) is by design, as each well represents an independent experimental unit containing multiple images (15 crops per well). This approach allows us to evaluate the model's performance at a biologically relevant scale, as compounds are administered and evaluated at the well level in actual drug screening applications.

We have clarified the above in the Figure 4 legends and “Ensemble-based batch effect mitigation” section in Result. We thank you for helping us improve the clarity of our methodology.

***Comment 32:***

The ensemble method adopted to mitigate batch effects is a strong feature of this study. However, additional clarification on the effectiveness of the leave-one-plate-out cross-validation approach would be beneficial. Specifically, statistical comparisons between model performance with and without this approach could provide quantitative support for its utility***.***

***Response to Comment 32:***

Thank you for suggesting that we quantitatively demonstrate the effectiveness of our ensemble approach. We have addressed this by adding statistical analysis and a new figure (Figure 5) to the manuscript. We used McNemar's test to perform comparisons between model performances with and without the ensemble approach. The results confirmed that the two models exhibited statistically significant differences in performance. Specifically, the observed data revealed that the ensemble-based model correctly classified 32 samples that the best model (without ensemble approach) misclassified, while the best model correctly classified only 1 sample that the ensemble-based model misclassified. In summary, the performance of the two models differs significantly, and it can be concluded that the ensemble-based model outperforms the best model.

***Comment 33:***

Including visualizations, such as t-SNE plots, to demonstrate how the ensemble approach reduces batch effect in feature space would make this approach clearer and support its validity.

***Response to Comment 33:***

Thank you for this valuable suggestion regarding visualization methods. While t-SNE plots can provide useful insights into feature space distributions, we chose to validate our ensemble approach through McNemar's test, which directly evaluates the statistical significance of performance differences between models. This test specifically assessed the disagreements between the best single-fold model and our ensemble-based model, revealing a statistically significant improvement (χ² = 27.27, p = 1.76×10⁻⁷) in classification performance with the ensemble approach. The test results, combined with the higher F1 score of the ensemble model (77% versus 69%), provide strong statistical evidence for the effectiveness of our approach in mitigating batch effects while improving classification performance. We believe this statistical validation offers a more direct and quantitative assessment of our ensemble method's performance compared to feature space visualizations.

We have addressed this by adding statistical analysis and a new figure (Figure 5) to the manuscript.

***Comment 34:***

There are a few grammatical errors on line 228 and 229. I suggest that the authors thoroughly read through the manuscript and make correct all errors.

***Response to Comment 34:***

We have corrected the errors. Thank you for this suggestion.

***Comment 35:***

I suggest that the figures in the paper should be enhanced for clarity.

***Response to Comment 35:***

We have enhanced the figures.

***Comment 36:***

I suggest that the authors number all the equations in the paper.

***Response to Comment 36:***

We have numbered all the equations.

***Comment 37:***

I suggest that the authors add a brief section discussing potential limitations, particularly related to dataset size, generalizability, and model complexity.

***Response to Comment 37:***

We have added the following as one paragraph to the discussion section.

“While our approach demonstrates significant potential, there are opportunities for further refinement. The mode’s ability to capture intermediate levels of inflammation could be enhanced to capture subtle transitions between cellular states. Increasing the dataset size could enhance generalizability across various experimental conditions, and modifying the model for other cell types might extend its applicability beyond primary cultured cells. Though our ensemble strategy effectively addresses plate-level effects, the approach introduced by this approach presents both advantages and challenges. While enhancing predictive power, it demands optimizing computational efficiency to further support hight-throughput screening. ViT demonstrate superior capability in detecting global dependencies within images, suggesting that with larger datasets, understanding of complex relationships between cells could significantly improve. Therefore, although ViT models proved less optimal for our requirements, its their capabilities, combined with CNN models’ strength in local feature detection, point to promising direction for future work. Studies could leverage hybrid architectures that integrate the complementary proficiency of CNN and ViT models to enhance sensitivity to both morphological changes and intercellular relationships.”

***Reviewer #4:***

***Comment 38:***
The manuscript’s description of “enhancing understanding” and “streamlining processes” remains vague, with no clear indication of how these improvements quantitatively advance current methodologies. Furthermore, there is limited information on how the DL model was optimized for detecting morphological phenotypes specific to neuronal and glial cells, especially given the inherent complexity and variability within CNS cell types. Finally, the study lacks rigorous validation steps or comparative analysis with existing methods, raising questions about the reproducibility and robustness of the findings. ***Response to Comment 38:***
Thank you for these important points about the need for more specific quantification and validation of our methodology.

We have Added statistical comparison (McNemar's test) demonstrating significant improvement of our ensemble approach over single-model methods (Fig 5).

For detecting morphological phenotypes specific to neuronal and glial cells, we used primary cultures of brain cells rather than cell lines to better represent real biological states. Training data included varying degrees of inflammation induced by different LPS concentrations, helping the model learn subtle morphological transitions. Primary cultures maintain the intrinsic heterogeneity and complex morphological features of CNS cells. This approach ensures the model learns from physiologically relevant cellular phenotypes. We have added the brief discussions about limitation and future studies to our manuscript.

***Reviewer #5:***

***Comment 39:***

The methods were validated with cross validation but the authors didn't discuss how would such system be deployed for real-world screening especially on the compute resource requirement.

***Response to Comment 39:***
Thank you for this suggestion. We intentionally limited our cross-validation to 4 groups instead of 7 to balance effectiveness with efficiency. This reduced computational overhead while maintaining ability to capture plate-to-plate variations. Our system is designed to work with standard laboratory imaging equipment. The pipeline can be easily integrated with existing high-throughput screening workflows.

***Comment 40***:
The exploration of data augmentation to address the batch effect is lacking, the use of ensemble method is useful but other approaches can be explored as part of the comparison.

***Response to Comment 40:***
Thank you for this suggestion. We acknowledge that while our ensemble method proved effective, other approaches could have been investigated. However, we focused on the ensemble method as it effectively mitigated batch effects while maintaining computational efficiency. Our approach showed significant improvement (Fig 5). We agree that other techniques could be explored for future study.

***Comment 41***:
Model interoperability study is also missing, the authors can explore how to interpret the proposed CNN based model and highlight the feature importance through attribution via methods such as saliency and guided gradient.

***Response to Comment 41:***
A detailed analysis of feature maps (Supplementary Fig. 3, 4) revealed that it was sufficient to identify the key regions of interest prioritized by the model without requiring additional interpretability analyses such as saliency maps or guided gradients. Notably, for microglial cells, the model effectively captured the activated regions, demonstrating its capability to focus on the relevant morphological features critical for classification.

***Comment 42:***
The integration of other real-time phenotypes in addition to imaging input can potentially improve model capacity.

***Response to Comment 42:***
Thank you for this insightful suggestion about integrating additional phenotypic data. We agree that incorporating multiple phenotypic readouts (gene expression, biomarkers…) could enhance our model's predictive capacity. Such integration would allow us to capture both structural and functional changes in cellular states, potentially improving the model's predictive power and biological relevance. These improvements will be addressed in subsequent research
