# Supplementary material for: Unveiling CNS cell morphology with deep learning: A gateway to anti-inflammatory compound screening
Source: PLoS One. 2025 Mar 21;20(3):e0320204. doi: 10.1371/journal.pone.0320204 (PMC11927906; doi:10.1371/journal.pone.0320204)
Supplement: Attachment — Submitted filename: Review Comments.docx [file pone.0320204.s006.docx]

This manuscript presents a promising DL-based framework for CNS cell morphology analysis in drug screening. The manuscript is well written. However, with clarifications on the methodology, additional visualizations, and expanded discussions on applications and limitations, this work could make a strong contribution to the field. Here are some comments and suggestions to enhance the quality and impact of the work:

1. The manuscript discusses the use of CNN and ViT for classifying inflammatory states in CNS cells. While EfficientNet-B5 is selected as the optimal model, the criteria and reasoning behind the preference over ViT models could be further clarified. Including more detailed comparisons, particularly around model interpretability and applicability for CNS cell morphology, could enhance the reader’s understanding.

2. How many images are in the training and test set? I suggest that the authors clearly state the number of training and test samples and the train-test-split ratio.

3. The confusion matrix in Figure 3 is not very clear. Which model is used to generate the plot and why are there few sample sizes?

4. The ensemble method adopted to mitigate batch effects is a strong feature of this study. However, additional clarification on the effectiveness of the leave-one-plate-out cross-validation approach would be beneficial. Specifically, statistical comparisons between model performance with and without this approach could provide quantitative support for its utility.

5. Including visualizations, such as t-SNE plots, to demonstrate how the ensemble approach reduces batch effect in feature space would make this approach clearer and support its validity.

6. There are a few grammatical errors on line 228 and 229. I suggest that the authors thoroughly read through the manuscript and make correct all errors.

7. I suggest that the figures in the paper should be enhanced for clarity.

8. I suggest that the authors number all the equations in the paper.

8. I suggest that the authors add a brief section discussing potential limitations, particularly related to dataset size, generalizability, and model complexity.
